# How should we talk to pregnant women about physical activity? A scoping review of physical activity during pregnancy communication by healthcare professionals in the UK

**DOI:** 10.1080/21642850.2025.2581351

**Published:** 2025-11-23

**Authors:** Chloë Williamson, Graham Baker, Marlize De Vivo, Hayley Mills, Linda Bauld, Rebecca M. Reynolds, Anna Boath, Paul Kelly

**Affiliations:** a Physical Activity for Health Research Centre, University of Edinburgh, Edinburgh, UK; b Advanced Wellbeing Research Centre, Sheffield Hallam University, Sheffield, UK; c The Active Pregnancy Foundation, England, UK; d Faculty of Science, Engineering and Social Sciences, School of Psychology and Life Sciences, Section of Sport, Exercise & Rehabilitation Sciences, Canterbury Christ Church University, Kent, UK; e Usher Institute and Behavioural Research UK, University of Edinburgh, Edinburgh, UK; f Centre for Cardiovascular Science, Queen's Medical Research Institute, University of Edinburgh, Edinburgh, UK

**Keywords:** Exercise, guidance, midwifery, pregnant, maternal, communication

## Abstract

**Introduction:**

Research is needed to provide healthcare professionals (HCPs) with guidance on how to best communicate with pregnant women about physical activity (PA). This study aimed to answer: what is known about PA communication between HCPs and pregnant women in the UK?

**Methods:**

Design: scoping review, adhering to established guidance (including the PRISMA-ScR). Research questions were informed by the Physical Activity Messaging Framework (PAMF). Relevant studies were identified by searching electronic databases and contacting key stakeholders. All studies were double screened. The data extracted included findings related to concepts within the PAMF. The results were presented in a descriptive numerical analysis and a narrative summary.

**Results:**

Twenty-one studies were included. 81% were from England, and 71% involved solely qualitative methods. HCPs lack the knowledge and time required to provide PA advice, and stigma around weight prevents communication. Pregnant women feel that PA during pregnancy is dangerous and have low knowledge of the guidelines and benefits of PA. Many do not receive PA advice from HCPs, and where they do, it is minimal, contradictory, confusing, inconsistent, and negative. Tailored information and clear advice on what is safe, delivered using non-judgemental tones is desired. Pregnant women view HCPs as important messengers.

**Conclusions:**

HCPs should address the perception that PA is dangerous by communicating relevant information from PA guidelines and highlighting wide-ranging benefits of PA during pregnancy (including benefits to mental health). Communication should be non-judgmental, clear and consistent. HCPs should be supported and trained to provide PA advice as part of their role.

## Introduction

Physical inactivity is a leading cause of noncommunicable disease worldwide (Lee et al., 2012), and its prevalence is increasing globally (Strain, 2024). Finding ways to improve population-level physical activity (PA) is a public health priority, with a particular focus on reducing the inequalities required (Strain, 2024). The prevalence of physical inactivity is higher among females than among males (Strain, 2024), making them a priority target for PA promotion efforts.

PA (including structured exercise) plays an important role throughout life, including during pregnancy, with benefits such as improved cardiorespiratory fitness, decreased risk of preeclampsia, gestational diabetes *mellitus* (GDM), caesarean delivery, excessive gestational weight gain, hypertensive disorders, anxiety, prenatal depression, and fewer newborn complications (e.g. small/large for gestational age or birth injury) (Andargie et al., 2025; Department of Health & Social Care, 2019; Evenson et al., 2009; Gonzalez, 2024; Kieffer et al., 2002; Ribeiro et al., 2021; Xie et al., 2024). PA benefits in the postpartum period are also documented, including reduced depression, improved emotional wellbeing, and a restoration of prepregnancy weight (Department of Health & Social Care, 2019; Dipietro, 2019). The World Health Organization (WHO) and the UK Chief Medical Officers (CMOs) recommend at least 150 minutes of moderate-intensity PA per week, as well as muscle strengthening activities (World Health Organization, 2020) (UK guidelines specify twice per week (Department of Health & Social Care, 2019) during pregnancy and postpartum. During pregnancy, those who are already active before pregnancy are encouraged to continue, and those who were previously inactive are recommended to start gradually. Both sets of guidelines also provide safety considerations, e.g. avoiding exercising in extreme heat and avoiding ‘bumping the bump’ (Department of Health & Social Care, 2019; World Health Organization, 2020).

However, evidence suggests that PA levels decline during pregnancy (Evenson et al., 2009; Gonçalves et al., 2024; Kieffer et al., 2002; Pebley et al., 2022; Versele et al., 2023), with pregnant women on average being less physically active than non-pregnant women (Gaston & Cramp, 2011; Laudańska-Krzemińska & Krzysztoszek, 2024). While some reduction in PA during pregnancy is typical and indeed potentially expected (due to, for example, pregnancy-related nausea or a lack of time or energy to engage in PA) (Morris et al., 2020; Reyes et al., 2013), promoting regular and appropriate PA in this population is important for a healthy pregnancy as discussed. Specific populations, for example, those with overweight or obesity, can be targeted for interventions during pregnancy (Dodd et al., 2014), and the antenatal period has been described as a ‘teachable moment’, particularly in the context of weight management, extending across pregnancy and postpartum and longer-term obesity prevention (Phelan, 2010). Antenatal care is therefore one potential route for promoting PA during pregnancy, but further research is warranted to develop universal (i.e. non-weight-focused) interventions to promote PA widely.

The WHO describes antenatal care as ‘*care provided by skilled healthcare professionals (HCPs) to pregnant women in order to ensure the best health conditions for mother and babies*’ (World Health Organization, 2016). The use of health communication by HCPs, which can target individual and societal factors related to PA (Butland, 2007; Milton et al., 2021; World Health Organization, 2019), aligns with guidance from both the WHO and the National Institute for Health and Care Excellence (NICE) in the UK (World Health Organization, 2016). This is important, as while *physical* barriers to PA are faced during pregnancy, *individual* and *societal* barriers such as a lack of knowledge of what PA is safe, the opinions of others in social circles (McKeough et al., 2022), fear of miscarriage, and lack of access to consistent information and support from HCPs (Gonçalves et al., 2024) are also important factors in determining behaviour. Communication strategies promoting PA and exercise can address these barriers to alleviate concerns and facilitate engagement.

The potential impact of communication approaches is demonstrated in recent evidence from Canada, which found that including PA education by prenatal nurses and PA prescription by physicians is associated with positive outcomes, including a 29% lower odds of developing gestational weight gain, 73% lower odds of developing gestational hypertension, and 40% lower odds of being large for gestational age (Saidi et al., 2023). Despite the promise of PA communication as an approach, there is a scarcity of research focusing specifically on HCP PA communication during pregnancy, with the research that does exist focusing largely on maternal obesity and the management of gestational weight gain (Heslehurst et al., 2014).

The UK CMOs' PA Guidelines Communications Framework (Department of Health and Social Care, 2023) aims to support the communication of guidelines to professional audiences, including HCPs. This framework acknowledges that HCPs are a key conduit through which messages to the public can be delivered (Department of Health and Social Care, 2023). However, there is still a need to provide HCPs with guidance on how to best communicate about PA. Research concerning public-facing PA communication for pregnancy has been highlighted as a gap (Williamson et al., 2020), and specific recommendations for the communication of pregnancy PA guidelines (such as those from the WHO and UK CMOs) and related information by HCPs in the UK do not exist. Although several reviews have explored HCP communication during pregnancy, they have focused predominantly on obesity and weight-related communication (Heslehurst et al., 2014; Nagpal et al., 2020; Weeks et al., 2018) rather than PA-focused communication.

To develop communication recommendations and facilitate widespread dissemination of guidelines for all pregnant women in the UK, there is a need to review the existing evidence on PA communication by HCPs during pregnancy. Therefore, this review aimed to understand what is known about PA communication between HCPs and pregnant women in the UK.

## Materials and methods

### Design

To accommodate the broad nature of our aim and allow the inclusion of several evidence types, a scoping review was deemed the most appropriate design (Grant & Booth, 2009). To enhance robustness, we adhered to an established five-step protocol (Arksey et al., 2005), the Preferred Reporting Items for Systematic Reviews and Meta-Analyses extension for scoping reviews (PRISMA-ScR) (see checklist in Appendix A) (Tricco et al., 2018), and methodological guidance for the conduct of scoping reviews (Peters et al., 2020).

### Stage 1: identifying the research question(s)

The Physical Activity Messaging Framework (PAMF) (Williamson et al., 2021a) was used to shape research questions (RQs) and guide data extraction and analysis. The PAMF is divided into three key sections that consider (i) message aims and context, (ii) message content, and (iii) message delivery. One of the specified uses of the framework is to provide a categorisation tool for literature reviews in the area of PA communication (Williamson et al., 2021b). Thus, to address the overall RQ, three specific RQs directly aligning with the three sections of the PAMF were developed. These RQs and how each corresponds with the PAMF are outlined in Table 1.

**Table 1.** Explanation of PAMF-informed research questions.

PAMF section	Relevant key concepts for this review	Corresponding research question (RQ)
Message aims and context	This section of the PAMF encompasses concepts relating to the target audience, message aims, and contextual considerations. The target audience in this situation is predefined (pregnant women). However, this review can explore important contextual factors (e.g. population-specific barriers) to help identify potential messaging aims for this population (e.g. address lack of knowledge or improve confidence).	RQ1: what is known about important contextual factors for PA communication from HCPs during pregnancy?
Message content	This section encompasses various concepts related to the content of messages, for example, the type of information pregnant women receive, the way messages are framed (positively or negatively), the use of tailoring and targeting, and the language, tone and choice of words used by HCPs.	RQ2: what is known about the content of PA communication by HCPs during pregnancy?
Message delivery	Concepts relating to message format and delivery relevant for this review include the way information is conveyed (e.g. face-to-face, telephone call, via app), who the messenger is (which HCP?), the volume of information and frequency of message delivery.	RQ3: what is known about how PA related information is communicated by HCPs during pregnancy?

### Stage 2: identifying relevant studies

Relevant studies were identified by (a) searching electronic databases (MEDLINE, SPORTDiscus (Ebscohost), Embase, ProQuest Social Science Journals, and PsycInfo); (b) contacting key academic, policy and practice stakeholders requesting information on relevant studies; and (c) hand-searching reference lists of key studies and checking recent publications by key authors. Searches were conducted for studies published from inception to 10 April 2024.

The search strategy was designed to be as comprehensive as possible for the resources available to identify primary sources (published and grey literature) and reviews (Peters et al., 2020). In the context of PA during pregnancy, advice is commonly coupled with dietary practices and may specifically reference weight status (i.e. body mass index) and gestational weight gain guidelines. For example, NICE guidance states that HCPs should have the communication skills required to approach the subject of weight management during pregnancy in a sensitive manner, providing practical advice on how to improve PA levels and diet (National Institute for Health and Care Excellence (NICE), 2010). To ensure that as many studies including data on PA communication as possible were identified, databases were searched for titles and abstracts that contained at least one ‘maternal pathway’ term, one ‘communication term’, as well as at least one ‘physical activity’, ‘diet’, or ‘weight’ term and one ‘healthcare professional’ term. While acknowledging differences in communication approaches (e.g. messaging, advice, guidance), this review took an inclusive approach and was not limited to one specific type. Appropriate truncation symbols and wild cards were used to account for search term variations and to maximise searches. The complete search strategy for MEDLINE can be found in Appendix B. The inclusion and exclusion criteria are shown in Table 2. Key stakeholders were also contacted (academic researchers, professional organisations and charities) and asked if they had any studies or reports on HCP communication around PA during pregnancy. Specifically, this involved contacting 16 individuals across the following 12 organisations: Office for Health Improvements and Disparities (formerly Public Health England), Royal College of General Practitioners, Public Health Scotland, Moving Medicine (Faculty of Sport and Exercise Medicine), Tommy’s, Diabetes UK, Kin Collective, Obesity Health Alliance, Royal College of Midwives, Royal College of Nursing, the SIGN Guideline Diabetes and Pregnancy 2021 update group, and Northumbria Healthcare NHS Foundation Trust.

**Table 2.** Inclusionand exclusion criteria.

Inclusion criteria	Exclusion criteria
Research conducted in healthy or clinical populations (e.g. pregnant women with Gestational Diabetes) Research conducted in pregnant women with or without overweight or obesity Research conducted with healthcare professionals Research on physical activity (of any type, including exercise and sport) communication by HCPs of any type Articles published in peer-reviewed journals and grey literature Articles reporting on development or effects of physical activity communication for pregnant women Articles published in English Articles focusing on UK populations Primary research studies of any design (including both quantitative or qualitative approaches)	Articles not focusing on pregnancy Articles not focusing on healthcare professional messaging or communication Articles without physical activity communication insights Articles not available in English Abstracts without full text Reviews* Studies based outside the UK Conference abstracts * Any relevant review studies were initially retained, and primary studies included in these reviews were appraised against the above criteria.

### Stage 3: study selection

All identified studies (including those from stakeholder analysis) were uploaded to Covidence™ review software (Veritas Health Innovation, Melbourne, Australia. Available at www.covidence.org, work completed 2024). Duplicates were automatically removed. All studies were double screened by two independent reviewers (CW, PK, GB, NH, BM, KS or AB) at both the (i) title and abstract and (ii) full-text stages, with conflicts resolved by a third researcher (Peters et al., 2020).

### Stage 4: data extraction

Data were extracted into a data extraction form using Excel (Supplementary Material). To ensure consistency, data from five studies were extracted independently (by CW, PK and GB) and compared. Discrepancies were discussed, and a consistent approach was agreed upon before the remaining studies were extracted. Data extracted included the following information:

- General study information, including author, title, year, study location.
- Study characteristics, including design, study aim, and study methods.
- Participant characteristics, including number of participants and who they were (pregnant women, HCPs, or both).
- Description of findings around PA communication relating to (i) contextual factors, (ii) message content or (iii) message delivery (i.e. relevant to any of the three RQs). Where relevant (e.g. in qualitative studies), this included the extraction of illustrative quotes.

### Stage 5: collating, summarising and reporting

A descriptive numerical analysis was conducted to provide insight into the extent, nature, and distribution of the evidence base. Basic coding of the extracted data from the included studies was then conducted to identify key themes related to each study RQ. These themes, mapped to RQs 1−3, were then used to produce a narrative summary (Peters et al., 2020).

## Results

### Descriptive numerical analysis

**Figure 1.**
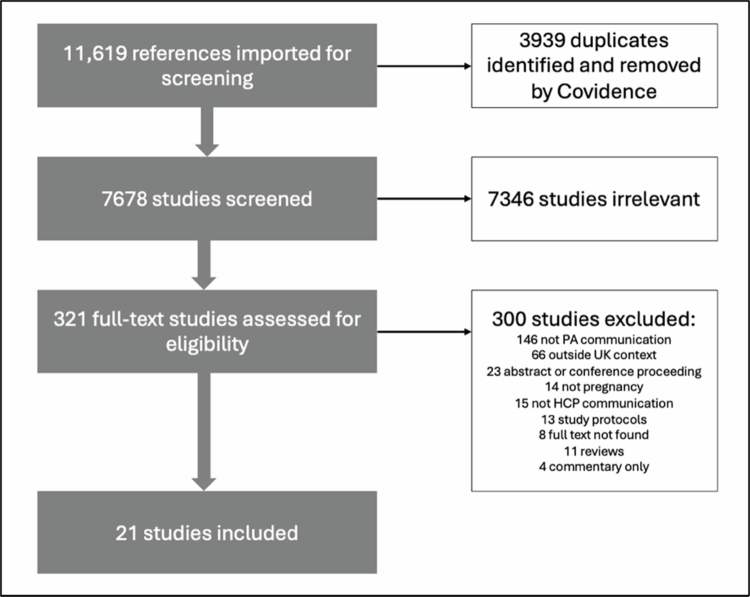
Study selection process (adapted from Covidence™).

A total of 11,619 records were imported to Covidence™ for screening (11,596 from database searches, 6 from stakeholder consultations and 17 from hand searching). Following duplicate removal and screening, 21 studies were included in the final analysis (see Figure 1). The full data extraction form is available in the Supplementary Material.

Of the 21 studies, 17 (81%) were in England, and two (9.5%) were in Scotland. The two remaining studies (9.5%) recruited participants from across the UK, using national public forums and charities. Studies were published between 2004 and 2021, mostly (*n* = 19, 90.5%) in peer-reviewed journal articles, with two (9.5%) from grey literature (one charity report and one commissioned report). Three (14%) of the studies were published from 2004–2010, ten (48%) from 2011–2015, seven (33%) from 2016–2020, and one (5%) from 2021 or later.

Among the 21 studies, most (*n* = 15, 71%) involved solely qualitative methods, primarily semi-structured interviews. Focus groups, online surveys, and analyses of internet forum posts were other qualitative methods used. Five studies (24%) used mixed methods, whilst one (5%) was quantitative. The quantitative methods applied included surveys. All studies (*n* = 21, 100%) were observational in nature, and there were no experimental designs. These studies aimed to explore, for example, women’s responses to advice provided about behaviour change during pregnancy, the types of information and advice given to pregnant women, and HCP experiences of providing PA advice.

Thirteen studies (62%) recruited women, either pregnant or having given birth recently, whilst six (28.5%) recruited HCPs only. Two studies (9.5%) involved both HCPs and women. HCPs were predominantly midwives, but some studies also involved HCPs such as health visitors, sonographers, obstetricians, and anaesthetists. Almost half of the studies (*n* = 10, 48%) focused specifically on women with overweight or obesity (or HCPs working with them).

### Narrative summary

**Figure 2.**
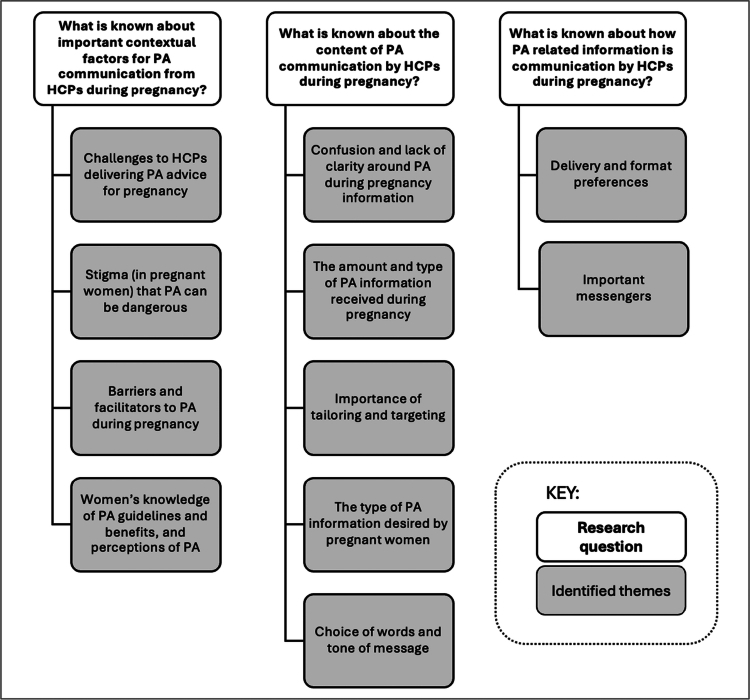
Overview of themes organised by research question.

This review identified several themes related to the overarching research question. These themes are presented below in a narrative summary organised by the three RQs. An overview of the themes is provided in Figure 2. Througout the below summary, the terms PA and exercise are used to reflect terms used in primary studies, acknowledging that exercise is a subtype of PA.

*Research question 1: What is known about important contextual factors for PA communication from HCPs during pregnancy?*

#### Challenges to HCPs delivering PA advice for pregnancy

Across multiple studies, midwives expressed that they lacked knowledge of what advice to provide (De Vivo & Mills, 2019; Smith et al., 2011), confidence (De Vivo & Mills, 2019), time to discuss PA (as they have several other topics to cover) (Dinsdale et al., 2016; Ells, 2018; Macleod et al., 2013; Smith et al., 2012), and awareness of local PA opportunities available to pregnant women (De Vivo & Mills, 2019). HCPs felt that they need more training on what PA-related advice to provide pregnant women with whom to support their PA behaviour (De Vivo & Mills, 2019; Ells, 2018; Heslehurst et al., 2015; Smith et al., 2012). Stigma around weight was also found to prevent midwives from discussing PA with pregnant women due to fear of causing offense or embarrassment (Ells, 2018; Furness et al., 2011; Lawrence et al., 2020; Macleod et al., 2013; Smith et al., 2012).

Another challenge was that HCPs may not be clear about their potential role in providing PA advice. In one study, midwives assumed that information on PA during pregnancy was already understood (Dinsdale et al., 2016), echoed by women in another study reporting that they felt that midwives assumed that they already knew about PA during pregnancy (Padmanabhan et al., 2015). Less than half (46%) of the midwives in one study reported that their job was to provide weight management advice to pregnant women with obesity (including advice on PA) (Macleod et al., 2013), and the midwives in another study reported feeling frustration around the responsibility of communicating about PA being passed between HCPs and exercise professionals (De Vivo & Mills, 2019).

#### Stigma (in pregnant women) that PA can be dangerous

This review identified several challenges faced by pregnant women themselves. First, across several studies (including clinical and non-clinical populations), the perception that PA during pregnancy is dangerous was evident (Findley et al., 2020; Gross & Bee, 2004; Lie et al., 2013; Tommy's, 2009). In one study, 68% of women indicated that during pregnancy, they perceived an unnecessary degree of risk associated with PAs they engaged in before becoming pregnant (Gross & Bee, 2004) and believed that aspects of their former PA behaviour could jeopardise progress of their pregnancy (Gross & Bee, 2004). In another study, women reported feeling a strong sense of social pressure to conform to others’ views of PA during pregnancy, e.g. colleagues, friends and family who view PA as dangerous during pregnancy (Findley et al., 2020). A further study found that this perceived risk of PA led women to prioritise other behaviours, such as nutrition, over PA during pregnancy (Padmanabhan et al., 2015). In support of this finding, HCPs in one study reported that pregnant women may presume that exercise is risky and acknowledged that reassurance may be required (Heslehurst et al., 2011). Also relating to this perceived risk, women had specific concerns about the safety of classes and suggested a preference to attend classes run by midwives or other trained HCPs (Padmanabhan et al., 2015), such as midwife-led walks (Furness et al., 2011).

#### Women’s knowledge of PA guidelines and benefits, and perceptions of PA

In relation to women's knowledge of PA and its benefits, this review found mixed findings. For example, a longitudinal mixed methods study found that only 26% of women rated ‘regular exercise’ as important during pregnancy (compared with, for example, 91% for ‘getting a good night's sleep’) (Gross & Bee, 2004). In another study, pregnant women were found to have low awareness of the wide-ranging PA benefits, with the most frequently reported benefit being ‘burning calories’ (Tommy's, 2009). Conversely, women in another study reported that being healthy and maintaining fitness during pregnancy were important (Findley et al., 2020).

In addition to maintaining fitness, the benefits of PA reported by pregnant women included both psychological and physiological benefits. Physiologically, women reported the belief that PA enables easier labour (Padmanabhan et al., 2015). Psychologically, women mentioned PA helping lift their mood (Findley et al., 2020), feeling more relaxed (Findley et al., 2020; Padmanabhan et al., 2015) and energised (Findley et al., 2020), allowing them to have fun (particularly with company), (Tommy's, 2009) and reducing loneliness (Lavender & Smith, 2016).

In relation to PA guidelines specifically, data were limited, but most of the women in one study were unaware of the intensity and level of PA required during pregnancy to gain benefits, and many overestimated the amount recommended in guidelines (Tommy's, 2009).

#### Barriers and facilitators to PA during pregnancy

In terms of facilitators, learning new forms of movement and setting personal goals (Lavender & Smith, 2016) and having support from and being active with others were viewed as facilitators to PA (Furness et al., 2011). Various physical, social, psychological and environmental barriers were identified. Physical barriers identified included tiredness and fatigue (Arden et al., 2014; 'Dinsdale et al., 2016; Eades et al., 2018; Findley et al., 2020; Lie et al., 2013; Tommy's, 2009) morning sickness (Arden et al., 2014; Findley et al., 2020; Tommy's, 2009), discomfort or pain (Dinsdale et al., 2016; Eades et al., 2018; Findley et al., 2020) and having a bump (Eades et al., 2018; Findley et al., 2020). Social barriers included caring responsibilities or demands for other types of childcare (Arden et al., 2014; Eades et al., 2018​​​​​​; Lie et al., 2013;​Tommy's, 2009), work pressures (Arden et al., 2014), having a busy life (Tommy's, 2009), and perceptions of friends and family (Lie et al., 2013). Several psychological barriers were also identified, namely, fears about the safety of the baby (Dinsdale et al., 2016; Findley et al., 2020), a lack of knowledge about safe exercise, guilt, a lack of motivation, and a lack of confidence (Tommy's, 2009). Finally, environmental barriers identified included the cost of PA (Dinsdale et al., 2016; Tommy's, 2009), a lack of appropriate options and facilities (Smith et al., 2011; Tommy's, 2009), not feeling safe (Tommy's, 2009), and weather (Eades et al., 2018; Tommy's, 2009).

*Research question 2: What is known about the content of PA communication by HCPs during pregnancy?*

#### Confusion and lack of clarity around PA during pregnancy information

Across multiple studies, women reported receiving contradictory (Gross & Bee, 2004), confusing (Arden et al., 2014; Furness et al., 2011; Gross & Bee, 2004) (e.g. around what is appropriate PA during pregnancy) (Eades et al., 2018), and inconsistent (Arden et al., 2014​​​​​​;​Findley et al., 2020; Furness et al., 2011) advice and messages from HCPs during pregnancy. One study reported that this resulted in women feeling unsure of whose advice to follow and left to ‘use common sense’ (Findley et al., 2020).

#### The amount and type of PA information received during pregnancy

The amount and type of PA-related information received appeared to differ across the studies included, and the results indicate that not all pregnant women receive advice on PA. In one study, only 64.3% of pregnant women reported receiving exercise or diet advice from an HCP (Brown & Amanda, 2012). In this same study, when discussing information received from HCPs, only 32.1% of the advice was about PA (compared to 67.9% being about diet) (Brown & Amanda, 2012). Another study found that more than half of the participants gained variable information from HCPs during pregnancy (Findley et al., 2020).

The data suggest that both HCPs and women perceived PA to be a low-priority topic of discussion. In one study, midwives referred to PA advice as a ‘tick box’ exercise that is not explored unless pregnant women raise it themselves (De Vivo & Mills, 2019). Only 15% of midwives from one study reported offering personalised advice regarding weight management based on PA levels (Macleod et al., 2013). Echoing this, women in other studies reported that PA related information was minimal (Padmanabhan et al., 2015; Brown & Amanda, 2012), was only provided when they sought it themselves (Dinsdale et al., 2016; Lawrence et al., 2020), and felt that NHS midwives did not instigate discussions around exercise (Heslehurst et al., 2015; Lawrence et al., 2020; Weir et al., 2010). In another study, almost all (96%) participants indicated that they had received advice regarding PA at least once during their pregnancy. However, only 49% of women in the same study reported that they received such advice on three or more occasions (Gross & Bee, 2004).

In studies where women did report receiving PA-related information, this included advice on safe exercise and avoiding over-exertion (Brown & Amanda, 2012), information about specific benefits and risks of different PAs (Gross & Bee, 2004), the importance of prenatal exercise (Gross & Bee, 2004), and, in one case, referral to a lifestyle programme (Lavender & Smith, 2016). Some data indicate that women felt information received was restriction-focused (Padmanabhan et al., 2015), more risk adverse than necessary (Findley et al., 2020), and that they received detailed information on what they should not do but no advice on what they should do (Padmanabhan et al., 2015).

Specifically, in women with GDM, nearly all women in one study recalled that they should reduce their future risk of type 2 diabetes via PA (Eades et al., 2018). However, women in this study felt that advice was brief and peripheral to the advice they received on their diet and that the vague and untailored information received left them frustrated (Eades et al., 2018). Conversely, women with GDM in another study were appreciative of the clinical support received and reported that advice helped them understand the role of PA in controlling blood glucose during pregnancy (Lie et al., 2013).

#### Importance of tailoring and targeting

In terms of tailoring, women reported desiring personalised advice (Arden et al., 2014) but felt that they were not given individually tailored advice (Brown & Amanda, 2012; Findley et al., 2020). This was in line with the fact that midwives reported feeling ill-equipped to deliver tailored content (De Vivo & Mills, 2019). Women stated the desire for midwives to ask questions and explore their perspectives rather than being left with the responsibility of raising issues themselves (Lawrence et al., 2020). In terms of targeting, the importance and possibility of delivering information based on four different ‘profile types’ involving consideration of previous PA levels and intention to be active during pregnancy was proposed in one study using theory of planned behaviour (De Vivo & Mills, 2021).

#### The type of PA information desired by pregnant women

This study found that women want clear advice on PA and exercise that is safe to take part in during pregnancy (Brown & Amanda, 2012; Dinsdale et al., 2016; Findley et al., 2020; Tommy's, 2009), practical advice (Arden et al., 2014) such as details of community exercise classes (Dinsdale et al., 2016), pregnancy-specific benefits of PA (Heslehurst et al., 2015), and clear and specific advice about how much PA is recommended (e.g. a specific number of minutes per day) (Heslehurst et al., 2015).

#### Choice of words and tone of message

Women wanted advice to be delivered sensitively (Arden et al., 2014), and felt that being 'told off' for certain behaviours left them feeling unsupported (Brown & Amanda, 2012). Women from one study reported feeling judged or under pressure to make a change (Lawrence et al., 2020). In other studies, pregnant women reported positive experiences of non-judgmental attitudes from HCPs (Furness et al., 2011), and others reported that they valued an ‘informal style’ of delivery (Lavender & Smith, 2016).

*Research question 3: What is known about how PA-related information is communicated by HCPs during pregnancy?*

#### Delivery and format preferences

Available data on delivery preferences are limited. Women in one study felt that face-to-face approaches would be most effective for the delivery of information but that receiving information in a leaflet or mini guide would also be practical and convenient (Tommy's, 2009). In another, women highlighted the importance of developing visual resources to help overcome literacy issues and suggested that the illustrations used provide an inclusive representation of the population being targeted (Ells, 2018).

#### Important messengers

In terms of important messengers or providers of information, women in one study felt that all HCPs had a responsibility to proveadvice and guidance on PA benefits, but that midwives were most appropriate for delivering this information (Weir et al., 2010). The importance of the HCP as a messenger was highlighted in a study in which pregnant women reported that the level of PA that they would be able to achieve during pregnancy was influenced by advice received from HCPs (Findley et al., 2020). However, in another longitudinal study, women reported that HCPs played a substantial role in disseminating advice at or before the 12-week mark but less so after that (Gross & Bee, 2004). In this same study, the primary sources of information reported were HCPs, books and magazines, and friends and family, with written information being particularly popular in early pregnancy (Gross & Bee, 2004).

## Discussion

### Summary of key findings

This review is the first to explore what is known about PA communication between HCPs and pregnant women in the UK. Of the included studies, we found that most of the research in this area is qualitative in nature, with more studies (over 80%) occurring in England than in other nations. Studies in this area involve both pregnant women and HCP participants. The principal findings include that HCPs lack the knowledge and time required to provide PA advice and that stigma around weight also prevents communication. Pregnant women feel that PA during pregnancy is dangerous and face several other physiological and psychological barriers to PA. Pregnant women also seem to have low knowledge of PA guidelines and limited awareness of the wide-ranging benefits of PA. Where women were aware of benefits, these benefits related predominantly to mental health (e.g. feeling relaxed and socialising). This review found that some pregnant women do not receive PA advice from HCPs, and where they do, it is minimal, contradictory, confusing, inconsistent, and negative. Pregnant women desire tailored information and clear advice on what is safe, delivered using non-judgemental language and tones. Finally, though not all HCPs may view offering PA advice as part of their role, pregnant women view them as important messengers.

### Comparisons with and contributions to literature

This review adds to several existing reviews related to HCP health communication during pregnancy. Another scoping review (Nagpal et al., 2020) summarized studies assessing potential causes of weight stigma in prenatal healthcare settings. In their recommendations, they suggest providing HCPs with sensitivity training to discuss obesity using a tailored approach and providing educational resources that explain potential risks. Though our findings also support providing HCPs with further training and offering educational resources on PA, the communication of risks of inactivity is not supported by our findings, potentially highlighting a key difference between weight-focused and universal communication. Rather, our findings support the promotion of PA benefits, particularly shorter-term benefits related to mental health.

There does appear to be agreement between some findings related to weight-focused and more universal PA communication. A narrative review (Weeks et al., 2018) summarised studies assessing gestational weight gain discussions between patients and HCPs to understand what is being exchanged. Like our review, they found communication to be infrequent and reported that patients received conflicting information between HCPs. The implications of this conflicting advice for guideline adherence warrant particular attention. When pregnant women receive contradictory messages about PA safety, they are left to use ‘common sense’ (Findley et al., 2020) and make their own judgements without adequate expertise. This creates several barriers to adherence. First, conflicting advice may reinforce the belief that PA during pregnancy is dangerous (Gross & Bee, 2004; Merkx et al., 2017; Tommy's, 2009), with women potentially interpreting contradictory messages as uncertain or lacking consensus among HCPs. Second, inconsistent messaging undermines trust in HCPs as reliable information sources, potentially leading women to avoid PA as a ‘safer’ option. Third, the burden of reconciling contradictory advice may be particularly challenging during pregnancy when women are already managing multiple information sources. Addressing this issue requires systemic change to ensure consistency across HCPs and contact points. Standardising training and providing readily available accessible resources based on current guidelines (Department of Health & Social Care, 2019; World Health Organization, 2020) and up-to-date messaging evidence could help ensure that women receive coherent and consistent evidence-based advice. Without such improvements, the guideline-behaviour gap is likely to persist.

Also with similar findings to our review, a systematic review (Heslehurst et al., 2014) included a meta-synthesis of HCPs' barriers and facilitators to implement pregnancy weight management guidelines. They found that HCPs generally lacked formal training (which was a barrier to practice) and that HCPs felt that other behaviour topics (smoking, breastfeeding) were more common on the national agenda. HCPs felt that good communication skills were important but lacked confidence and felt uncomfortable initiating conversations about weight. This highlights a need for changes that help move PA up on the communication priority list for HCPs.

Overall, the existing evidence base has a predominant focus on communication about weight with pregnant women with overweight and obesity. This is not surprising given the prevalence rates of obesity and its rightful status as a public health priority. However, all pregnant women can benefit from PA; thus, the importance of universal communication about PA during pregnancy should not be overlooked. Our findings complement many of the existing recommendations made by authors in the field yet build on this existing evidence by providing universal PA communication recommendations for HCPs to use when discussing PA with pregnant women, irrespective of weight status. These findings are relevant when promoting general PA or structured exercise. While delivery of tailored exercise programmes may require specialised advice, the fundamental communication principles identified here (clarity, consistency, non-judgmental tone) apply to both contexts.

A further finding that merits comment is that pregnant women in the UK may have low knowledge of PA guidelines and lack awareness of the wide-ranging benefits of PA. This is not surprising and reflects knowledge found in the general population, with surveys consistently reporting rates of knowledge of the UK CMO Guidelines of between 4% and 18% (Bromley et al., 2013; Hunter et al., 2014; Knox et al., 2013, 2015). Despite the knowledge of guidelines not necessarily translating to adherence to guidelines, if pregnant women are aware of guidelines and the benefits of PA during pregnancy, this may help address some of the specific barriers they face identified in this review and others, such as the perception that PA is unsafe and being unaware of how much and what type of PA to do. Efforts should therefore be made to effectively communicate the UK CMO PA Guidelines with pregnant women across the UK in a way that is meaningful for them, including providing information on the widespread benefits of PA during pregnancy.

### Implications for practice

PA guidelines for pregnancy from across the world have remarkable consistency with respect to what types and how much PA should be carried out and, importantly, the safety of PA (Hayman et al., 2023). The authors of a recent scoping review of PA guidelines for pregnancy concluded that ‘*the challenge now is to ensure that all who provide healthcare for women understand the guidelines and encourage safe participation in PA during pregnancy*’ (Hayman et al., 2023). Helping address this challenge, our research adds to a growing body of guidance available to those involved in communicating with various audiences about healthy behaviours. For example, the ‘Whole Day Matters’ Toolkit for Primary Care aims to assist primary care providers in Canada with promoting PA (Canadian Society for Exercise Physiology (CSEP), 2021), and the ‘Physical Activity: Applying All Our Health’ guide aims to help HCPs promote PA as part of daily practice in the UK (Office for Health Improvement and Disparities, 2022).

A recent qualitative evidence synthesis of practice recommendations across England NHS Trusts revealed that seven (of 29) of the identified weight management guidelines offered brief recommendations for PA, but overall, the information on how to translate this into pregnant women’s real lives was not evident (Goddard et al., 2023). Only three (of 29) guidelines recommended addressing women’s concerns around PA (Goddard et al., 2023). The findings of the current study therefore complement the existing guidance by providing specific guidance for HCPs when discussing PA with all pregnant women in the UK, regardless of weight status. In line with the UK CMOs' PA Guidelines Communications Framework (Department of Health and Social Care, 2023), our findings support the *This Mum Moves* cascade approach for delivering training in PA guidelines, which was found to improve the knowledge, confidence and professional practice of midwives and health visitors (Taylor et al., 2024).

Our findings highlight the importance of addressing key barriers to PA communication during pregnancy, such as HCPs’ lack of knowledge and time constraints. To mitigate these barriers, we recommend training that equips providers with the latest guidelines and communication techniques. For example, motivational interviewing is a communication technique that can encourage pregnant women to explore their motivations and address concerns through open-ended questions about pregnancies (Buchanan & Wilson, 2023). Additionally, integrating PA discussions into routine prenatal appointments and utilising digital resources can efficiently address time limitations. Leveraging facilitators such as the trust that pregnant women place in HCPs is also crucial. Encouraging peer-led exercise groups and ensuring consistency in messaging can enhance support for pregnant women. Basing communication on evidence and advocating for policy changes that allocate time for PA discussions can effectively address these barriers and capitalise on facilitators to promote healthful pregnancies.

This review sheds light on how PA guidelines can be communicated with all pregnant women. This is key, as currently, NICE guidelines on PA during pregnancy do not provide specific information on the frequency or duration of PA or emphasize muscle-strengthening PA, all of which are now present in global and national PA guidelines. The current findings can help bridge the gap between policy and practice by providing recommendations for the communication of PA in pregnancy-related information. Based on the findings of this review, recommendations for policy and practice, as well as example communication techniques that HCPs can use when discussing PA with pregnant women, are presented in Table 3.

**Table 3.** Recommendations for policy and practice.

Recommendations for HCPs when communicating with pregnant women (practice)	
Recommendation	Example phrases/guidance
Communications about PA should address the barrier that PA is perceived as dangerous by communicating that PA is safe, and providing specific examples of safe PA.	‘Activities like walking, swimming, and prenatal yoga are completely safe throughout pregnancy. They can help you feel more energised and relaxed.’
More effective communication of PA guidelines for pregnancy is needed (indeed, this may help address the barrier that PA is perceived as unsafe by pregnant women).	Break the 150-minute guideline message down into a daily, realistic target (e.g. approximately 30 minutes a day), highlighting that some is better than none. Explain clearly what counts as ‘moderate’ in a way they will understand and give relevant examples.
The wide-ranging benefits of PA for pregnancy should be communicated by HCPs, with particular focus on shorter-term mental and social health benefits.	‘Being active during pregnancy is a way to get fresh air, feel more relaxed, and connect with others who are going through the same journey.’
Where possible, tailoring communications to the individual could be beneficial (e.g. considering their unique situation).	‘I know you mentioned you are very tired. Even 10 minutes of walking could increase your mood and energy levels. What type of movement do you enjoy?’
PA advice should be provided using a nonjudgmental tone.	Open-ended questions such as ‘what would make it easier for you to move more?’ should be used to start a conversation rather than providing advice that could be perceived as judgemental, e.g. ‘you should exercise more.’
PA advice should be clear and consistent across HCPs.	All HCPs should reference the same guidelines and avoid contradictory messages about what is safe.
Recommendations for HCP service delivery training (policy and practice)	
HCPs are important messengers for PA advice and guidance during pregnancy. As per national guidelines, this should be highlighted to them in training and outlined as part of their role.	
HCPs should be educated on the importance of PA promotion. This training should cover clearly the evidence for PA benefits during pregnancy and dispel myths about PA being dangerous.	
Support to build capacity for PA communication to be included as part of the HCP role (particularly in midwives) in supporting pregnant women is needed. HCPs must have dedicated time in appointments for PA discussions and access to resources such as referral pathways.	
HCPs should be educated on PA benefits, as well as clear information on safe PA so that they can confidently and consistently communicate this with pregnant women. Training should include specific examples of safe activities and scripted language that addresses common concerns and barriers.	

HCP = healthcare professional

### Future research directions

This review found that the existing evidence base is predominantly qualitative. This is not a limitation, and indeed, qualitative approaches are suitable and helpful for exploring the aims of existing studies. Having said this, future research may wish to take empirical approaches to complement this largely qualitative evidence base. For example, experimental study designs (e.g. randomised controlled trials) with impact and outcome evaluations to address the effectiveness of PA communication for short-, medium-, and long-term outcomes (i.e. knowledge, motivation and behaviour) are recommended. However, consideration of ethics, context, and feasibility will be key in designing such studies. Experimental studies should use appropriate evaluative designs that are ethical and do not deprive pregnant women of important communication at a critical health stage, for example, by comparing usual care with enhanced messaging. The content of such interventions should also be informed by existing qualitative evidence and recommendations, such as those provided in this review.

Furthermore, future studies could explore if and how the perceptions and communication preferences of pregnant women differ by ethnic group, level of deprivation, disability or other long-term conditions given the disproportionate health burden to these groups, as a specific focus on this topic was not identified in this review. Further research may also be warranted to explore communication in pregnant people who do not identify as women. Finally, given that the existing evidence is England-centric, there is a need for research exploring PA communication in pregnant women across Scotland, Wales, and Northern Ireland.

### Strengths and limitations

This review adhered to established protocols and guidance to increase robustness. The findings provide clear recommendations to improve HCP communication about PA to pregnant women. However, there are limitations to address. First, the geographical scope. Although we chose to include only UK-based studies to provide context-specific recommendations, there are potentially lessons to be learned from other locations and settings that would complement these findings and further develop our understanding of HCP communication around PA during pregnancy. However, the findings of our scoping review are likely relevant for other countries with comparable healthcare systems, such as Australia and Canada. Our findings echo those from recent qualitative research in Australia, which revealed that discussions around healthy lifestyle behaviours between HCPs and pregnant women are often low in priority and inconsistent and are perceived as sensitive, making them difficult topics to raise (Knight-Agarwal et al., 2023).

A further limitation is that more than half the studies included in the review were conducted a decade ago or more. Although it is unlikely that the provision of information and advice about PA during pregnancy was transformed during that period, older studies may have less direct relevance to current practice. Additionally, we did not contact all possible stakeholders or search all relevant databases, such as CINAHL, which indexes nursing and midwifery literature. However, we believe that our comprehensive approach, including multiple health sciences and social sciences databases, stakeholder consultation, and reference list searching, captured the majority of relevant literature, particularly given the substantial overlap between databases and the fact that many key midwifery journals are indexed in MEDLINE and Embase.

Finally, this review was restricted to pregnancy only for feasibility and specificity reasons. Although it is expected that many of the recommendations presented here will be transferable to preconception and postnatal periods, further research is warranted to develop more specific recommendations and to support PA throughout these other key stages of the maternal pathway. This is important, as reproductive health should be considered as a continuum as opposed to isolated life stages.

## Conclusion

This study aimed to describe what is known about PA communication between HCPs and pregnant women in the UK, and to provide recommendations for HCPs when communicating with pregnant women, and for practice in relation to HCP training. We found that HCPs are seen as important messengers by pregnant women, despite not all HCPs feeling that providing PA advice is within their role. HCPs should aim to address the perception that PA is dangerous by communicating relevant information from the PA guidelines and highlighting wide-ranging benefits of PA during pregnancy, including benefits to mental health. HCPs' communication should be non-judgmental and should be clear and consistent across messengers. To support this, HCPs should be supported and trained to provide PA advice as part of their role to allow them to communicate about PA clearly and confidently with pregnant women. Future research is needed to evaluate the effects of PA communication by HCPs on various outcomes in pregnant women in the UK and to explore communication preferences in pregnant women from different ethnic groups, levels of deprivation, and those with disabilities or long-term conditions.

## Consent for publication

Not applicable.

## Supplementary Material

Supplementary material Appendix 3 Pregnancy review paper

## Data Availability

The authors confirm that the data supporting the findings of this study are available within the article and its supplementary materials. Full details of reviewed articles (data extraction sheet) are available as Supplementary Material.

## Appendices

### Appendix A. PRISMA-ScR checklist

Preferred Reporting Items for Systematic reviews and Meta-Analyses extension for Scoping Reviews (PRISMA-ScR) Checklist

**Table:**

Section	Item	PRISMA-ScR checklist item	Reported on page^#^
**Title**			
Title	1	Identify the report as a scoping review.	1
Abstract			
Structured summary	2	Provide a structured summary that includes (as applicable): background, objectives, eligibility criteria, sources of evidence, charting methods, results, and conclusions that relate to the review questions and objectives.	3
**Introduction**			
Rationale	3	Describe the rationale for the review in the context of what is already known. Explain why the review questions/objectives lend themselves to a scoping review approach.	4−6
Objectives	4	Provide an explicit statement of the questions and objectives being addressed with reference to their key elements (e.g. population or participants, concepts, and context) or other relevant key elements used to conceptualize the review questions and/or objectives.	6−7
**Methods**			
Protocol and registration	5	Indicate whether a review protocol exists; state if and where it can be accessed (e.g. a Web address); and if available, provide registration information, including the registration number.	
Eligibility criteria	6	Specify characteristics of the sources of evidence used as eligibility criteria (e.g. years considered, language, and publication status), and provide a rationale.	6
Information sources*	7	Describe all information sources in the search (e.g. databases with dates of coverage and contact with authors to identify additional sources), as well as the date the most recent search was executed.	6−7
Search	8	Present the full electronic search strategy for at least 1 database, including any limits used, such that it could be repeated.	6 (& Appendix/Additional file 3)
Selection of sources of evidence†	9	State the process for selecting sources of evidence (i.e. screening and eligibility) included in the scoping review.	7
Data charting process‡	10	Describe the methods of charting data from the included sources of evidence (e.g. calibrated forms or forms that have been tested by the team before their use, and whether data charting was done independently or in duplicate) and any processes for obtaining and confirming data from investigators.	7
Data items	11	List and define all variables for which data were sought and any assumptions and simplifications made.	7−8
Critical appraisal of individual sources of evidence^§^	12	If done, provide a rationale for conducting a critical appraisal of included sources of evidence; describe the methods used and how this information was used in any data synthesis (if appropriate).	
Synthesis of results	13	Describe the methods of handling and summarizing the data that were charted.	8
**Results**			
Selection of sources of evidence	14	Give numbers of sources of evidence screened, assessed for eligibility, and included in the review, with reasons for exclusions at each stage, ideally using a flow diagram.	8 (Figure 1)
Characteristics of sources of evidence	15	For each source of evidence, present characteristics for which data were charted and provide the citations.	9 (& additional file 3)
Critical appraisal within sources of evidence	16	If done, present data on critical appraisal of included sources of evidence (see item 12).	
Results of individual sources of evidence	17	For each included source of evidence, present the relevant data that were charted that relate to the review questions and objectives.	Additional file 3
Synthesis of results	18	Summarize and/or present the charting results as they relate to the review questions and objectives.	9−14
**Discussion**			
Summary of evidence	19	Summarize the main results (including an overview of concepts, themes, and types of evidence available), link to the review questions and objectives, and consider the relevance to key groups.	14−15
Limitations	20	Discuss the limitations of the scoping review process.	19−20
Conclusions	21	Provide a general interpretation of the results with respect to the review questions and objectives, as well as potential implications and/or next steps.	20−21
**Funding**			
Funding	22	Describe sources of funding for the included sources of evidence, as well as sources of funding for the scoping review. Describe the role of the funders of the scoping review.	21

JBI = Joanna Briggs Institute; PRISMA-ScR = Preferred Reporting Items for Systematic reviews and Meta-Analyses extension for Scoping Reviews.

* Where *sources of evidence* (see second footnote) are compiled from, such as bibliographic databases, social media platforms, and Web sites.

^†^ A more inclusive/heterogeneous term used to account for the different types of evidence or data sources (e.g. quantitative and/or qualitative research, expert opinion, and policy documents) that may be eligible in a scoping review as opposed to only studies. This is not to be confused with *information sources* (see first footnote).

^‡^ The frameworks by Arksey and O’Malley (6) and Levac and colleagues (7) and the JBI guidance (4, 5) refer to the process of data extraction in a scoping review as data charting.

^§^ The process of systematically examining research evidence to assess its validity, results, and relevance before it is used to inform a decision. This term is used for items 12 and 19 instead of ‘risk of bias’ (which is more applicable to systematic reviews of interventions) to include and acknowledge the various sources of evidence that may be used in a scoping review (e.g. quantitative and/or qualitative research, expert opinion, and policy document).

From: Tricco AC, Lillie E, Zarin W, O'Brien KK, Colquhoun H, Levac D, et al. PRISMA Extension for Scoping Reviews (PRISMAScR): Checklist and Explanation. Ann Intern Med. 2018;169:467–473. HYPERLINK "http://annals.org/aim/fullarticle/2700389/prisma-extension-scoping-reviews-prisma-scr-checklist-explanation" doi: 10.7326/M18-0850.

## Appendix B

### Example search (EMBASE)

**Table:**

1.	(maternal* or maternit* or pregnanc* or ‘pregnant women’ or pre-conception or inter-pregnancy or gestation* or ‘new mother’ or ‘new mum’ or antenatal or perinatal or prenatal or postnatal or postpartum or pre-pregnancy).tw.
2.	(messag* or persua* or communicat* or media* or education* or guideline* or advice or ‘brief advice’ or framing or framed or gain-framed or loss-framed or tailor* or target* or individualis* or marketing or therapy or counselling or ‘social support’).tw.
3.	(‘health care professional*’ or ‘healthcare professional*’ or HCP hospital* or doctor or GP* or ‘general practitioner*’ or nurse* or ‘health visitor*’ or midwife or midwives or care or ‘health professional*’ or professional or practice or ‘healthcare provider*’ or gynaecologist* or gynecologist* or OB-GYN* or obstetrician* or appointment*).tw.
**4.**	(nutrition* or diet* or food* or ‘dietary pattern*’ or ‘dietary factor*’ or fruit* or vegetable* or meat* or vitamin* or carbohydrate* or fish or protein* or ‘dietary supplement*’ or carbohydrate-restricted or fat-restricted or ‘caloric restriction’ or ‘diet therapy’ or ‘diet intervention’ or macronutrient* or beverage* or meal*).tw.
**5.**	(‘physical activit*’ or activ* or exercise* or sport* or fitness or yoga or pilates or walk* or cycl* or step* or lifestyle*).tw.
**6.**	(‘body mass index’ or BMI or obes* or weight* or ‘healthy weight’ or overweight or ‘body fat*" or ‘waist circumference*’ or ‘body composition*’ or ‘energy balance’ or ‘weight gain*’ or ‘weight loss*’ or ‘weight change*’ or ‘body weight trajector*’ or ‘postpartum weight retention’).tw.
**7.**	1 and 2 and 3
**8.**	4 or 5 or 6
**9.**	7 and 8
**10.**	limit 9 to (English language and full text)
