# Supplementary material for: How should we talk to pregnant women about physical activity? A scoping review of physical activity during pregnancy communication by healthcare professionals in the UK
Source: Health Psychol Behav Med. 2025 Nov 23;13(1):2581351. doi: 10.1080/21642850.2025.2581351 (PMC12646094; doi:10.1080/21642850.2025.2581351)
Supplement: Supplementary material — Appendix 3 Pregnancy review paper [file RHPB_A_2581351_SM3557.docx]

**Appendix 3: Data extraction of included studies**

| **Citation and country** | **Study design and data type** | **Participants** | **Study aim** | **Study methods** | **Summary of findings with coding relevant to RQs 2 and 3** | **Contextual factors coded for RQ1** |
| --- | --- | --- | --- | --- | --- | --- |
| Gross, H. and P.E. Bee, *Perceptions of effective advice in pregnancy – The case of activity.* Clinical effectiveness in nursing, 2004. **8**(3): p. 161-169.  England | Longitudinal mixed methods. Quantitative and qualitative. | N=57 pregnant women (“low-risk” pregnancy) | To use qualitative and quantitative data arising from a longitudinal study of pregnant women to examine women's responses to advice provided about changing their behaviour during pregnancy. | 57 volunteers were recruited via an East Midlands City Centre teaching hospital antenatal clinic. Over nine months, a semi-structured questionnaire and interview schedule collected data, alongside a range of psychological measures and activity measurement (such as actimetry). | At each visit, respondents were asked if they had received any advice regarding PA behaviour in 4 weeks prior to interview. **Percentage of women who responded positively to this question decreased over time (from 80% at 12 weeks to 53% at 38 weeks), but virtually all participants (96%) indicated they had received such advice at least once during the course of their pregnancy. 49% of women reported that they had received advice on 3 or more occasions**. ***The primary sources of this information were books/magazines, HCPs, and friends & family.   In early pregnancy, reading written advice was popular - 90% reported undertaking some form of reading at 12 weeks (books/magazines, etc).*  Health professionals played a substantial role in disseminating advice at or before 12 weeks of pregnancy, but less so thereafter.   The nature of the advice that was received centred on two main aspects: (1) Informing participants about specific benefits and risks of different activities, and (2) the importance of prenatal exercise and home stretching. Advice on routine daily activity was not recalled**. **The information received was often recalled as confusing or negative.**   The extent to which participants followed advice seemed to **depend on the clarity and applicability of information they were given.**   Approximately one in five women reported that they had often be**en met with confusing and contradictory recommendations.**  **68% of the sample indicated that during pregnancy they perceived an unnecessary degree of risk associated with some of the activities they had done before becoming pregnant. 75% of these believed an aspect of their former behaviour could directly jeopardise progress of their pregnancy.** | **Barrier to PA: perceptions of friends and family**  **81% of women perceived getting a good night's sleep as 'very important' behaviour during pregnancy. 'resting and relaxing' was also deemed important. Exercise regularly was only rated as important by 26% of women.** |
| Tommy's, Helping Obese Women to manage their weight in pregnancy. 2009: Colton House, Princes Avenue, London N3 2DB.  England | Qualitative | 17 women (14 White British; 14 currently pregnant; 15 age 25-35) from either London or West Midlands | This research aimed to identify what might help or hinder obese pregnant women in managing their weight through diet and exercise, to understand their attitudes, beliefs and behaviour in relation to diet and exercise during pregnancy, and assess information and support needs on this subject and how this can best be provided. | Interviews - either individual (5), paired friendship (3 x 2) or small groups (2 x 3). Recorded but no analytical approach reported. | **Women reported that knowing which exercises were safe for them to do during pregnancy would motivate them**. **They also reported that having fun (particularly by having company) during exercise would motivate them and provide them with support.**  **Many women felt face to face approaches would be most effective for delivery of information**, either one to one or in groups. The face to face format of delivery allows opportunity for questions and this eases anxiety.  **Receiving information in a leaflet or mini guide was deemed to be practical and convenient.**    **The use of happy, healthy looking and attractive larger women in visuals provided by Tommy's were well received - this helped them connect with the image. Seeing women that looked like them also helped stimulate desire to exercise.** | **Pregnant women in this research had low levels of awareness of the benefits of regular exercise. The most frequently reported benefit was burning calories.**   **Most women were unaware of the intensity and level of PA required to make a 'difference'. Most overestimated the recommended amount**  **Knowledge about exercising safely during pregnancy was limited, and exercise options were seen as limited.**   **Safety concerns about exercising while pregnant were evident.**   **Practical barriers like childcare, paying for classes and having an already busy life were also identified.** |
| Weir, Z., et al., Physical activity in pregnancy: A qualitative study of the beliefs of overweight and obese pregnant women. BMC Pregnancy and Childbirth, 2010. 10(1): p. 18-18.  England | Qualitative | 14 women in late pregnancy, 8 were obese, from Northeast England | This paper aimed to provide qualitative insights into how overweight and obese pregnant women living in the UK feel about physical activity in pregnancy within the context of their day to day lives. | Semi structured in depth interviews were conducted with 14 overweight and obese pregnant women. | **Women felt they had not had adequate levels of information, support, or advice regarding PA in pregnancy**.  **Women felt all HCPs had responsibility to provide advice and guidance about PA benefits, they felt the midwives were most appropriate to provide this advice.**  **Women felt that midwives did not actively encourage women to be physically active during pregnancy**. | ***Women were often recipients of lay knowledge and expertise from family members and partners, and this was often conflicting (some encouraging PA some discouraging it).   Study participants accessed media based pregnancy information (e.g., TV, website, books), but felt information accessed was negative, conflicting and impersonal.***   **Women cited several internal and external barriers to PA during pregnancy, including personal health problems (sickness, lack of energy) and lack of self-confidence and motivation.   Other barriers cited: work, lack of time, lack of childcare, guilt, lack of suitable exercise classes, not feeling safe, weather and money.** |
| Furness, P.J., et al., Maternal obesity support services: A qualitative study of the perspectives of women and midwives. BMC Pregnancy and Childbirth, 2011. 11(1): p. 69-69.  England | Qualitative | 6 obese pregnant women (BMI ≥30) and 7 midwives delivering maternity services in Doncaster, UK. Specific demographic data were not collected, but observations suggest all pregnant women who took part were White, and aged 18-40. | To explore women's experiences of managing weight in pregnancy and perceptions of women, midwives and obstetricians of services to support obese pregnant women in managing their weight. | Two semi-structured focus groups were used to collect data in a permissive and comfortable environment. Women and midwives were in separate focus groups. Focus group data were analysed using inductive thematic analysis. | **Obese pregnant women understood that activity was related to weight and health**, **but lacked confidence about appropriate levels and types of exercise in pregnancy**.   ***Confusion is exacerbated by ever-changing media messages****.*   **Midwives feel that clients lacked the knowledge and skills to maintain a 'healthy lifestyle**'.   **Stigma around obesity prevents midwives from addressing this via communication, with a fear of causing offense or upsetting**.   **Women reported receiving inconsistent messages about weight gain and weight management**.   **Women reported positive experiences of non-judgemental attitudes from HCPs** and provision of PA programmes.   **Midwife-led walks was an idea that was well received by the women** in this study. | Obese **pregnant women reported struggling to find the motivation to exercise.**   Pregnancy and motherhood were considered theoretically good opportunities to support obese pregnant women with behaviour change due to potential triggers such as struggling to carry baby upstairs etc.   Women reported feeling lonely and isolated at times. Inactivity seemed more likely when they were alone, **but others' support helped motivate them to be more active**.   Women preferred classes alongside other mothers because everyone's in the same boat. |
| Smith, S.A., et al., Community-based service provision for the prevention and management of maternal obesity in the North East of England: A qualitative study. Public health (London), 2011. 125(8): p. 518-524.  England | Qualitative | N=30 community service providers (including HCPs such as e.g., midwives, managers, family support workers, GPs, health visitor) | To develop a better understanding of community-based initiatives relating to maternal obesity, and to gain community service providers' views on maternal obesity services and their perceived role in the management and prevention of maternal obesity. | Semi-structured interviews and focus groups conducted with community service providers in Northeast of England. Thematic content analysis was used. | A lack of understanding amongst service users about physical activity was identified.   **There was a lack of knowledge among service providers around the safety of advising women to exercise.** | **Barrier to PA was identified as a lack of appropriate facilities (e.g., exercise classes with creche in same premises).** |
| Heslehurst, N., et al., How can maternity services be developed to effectively address maternal obesity? A qualitative study. Midwifery, 2011. 27(5): p. e170-e177.  England | Qualitative | N=30 maternity unit health care practitioners with personal experience of maternal obesity services. | To identify developments in maternal obesity services and health-care practitioners' views on how maternity services need to be further developed to be more effective in the care of obese pregnant women. | Semi structured interviews and focus groups were carried out with HCPs representing each NHS trust in the region that provides maternity services to identify views on the barriers, facilitators, advantages, disadvantages of developing maternal obesity services, and how maternity services can be more effective in managing maternal obesity. | Some HCPs thought that pregnant women **may presume that exercise was risky, and that reassurance may be required.** | The issue of obese women feeling comfortable attending general PA sessions was raised, or whether targeted sessions would be more appropriate. |
| Brown, A. and A. Amanda, Healthy weight management during pregnancy: what advice and information is being provided: Healthy weight management during pregnancy. Journal of human nutrition and dietetics, 2012. 25: p. 378-387.  UK National. | Cross sectional mixed methods survey – quantitative and qualitative | 59 women with singleton pregnancies and members of the National Childbirth Trust. Most participants were normal weight (55.9%) or overweight (25.4%) according to BMI. | To explore the information and advice given to pregnant women of different pre-pregnancy body mass index classifications (underweight, normal weight, overweight and obese). | Participants completed a survey that explored weight monitoring experiences and the diet and exercise advice received. Quantitative data were compared using Mann-Whitney U-tests and chi-squared tests. Qualitative data were analysed by thematic content analysis. | Only **64.3% of women reported receiving diet and exercise advice from a healthcare source**. Other advice came from magazines, books, friends and family and the Internet, and fitness advisors / gyms.   Relating to information received only from healthcare sources, **only 32.1% of the advice was about exercise as opposed to 67.9% being about diet.** Advice reported to cover **safe exercise, continuation of regular exercise, and avoidance of over-exertion or taking up new sports.   Several participants felt they were not given advice which was individually tailored:** "...it would be better to tailor antenatal sessions to the Mum focusing on their background and current situation... the method is appears to be now... it's very impersonal".   Participants highlighted they would like more emphasis and advice on exercise **which is safe during pregnancy**: "I was keen to keep up with exercise so asked and was just told it was okay to do so.. personal trainer at gym gave me a lot more detail... much more useful and safe advice"  Women felt they **received too brief / little advice from healthcare professionals**: "I feel the advice about exercise from the GP and midwife as very brief... nor did they explain why exercise should be moderate which would have made it more meaningful and understandable"   Several women felt a lack of support from healthcare professionals, "**The guy who gave me my 12-week scan began the consultation by telling me off for riding a bicyle while pregnant"** |  |
| Smith, D.M., A. Cooke, and T. Lavender, Maternal obesity is the new challenge; a qualitative study of health professionals’ views towards suitable care for pregnant women with a Body Mass Index (BMI) ≥30 kg/m2. BMC Pregnancy and Childbirth, 2012. 12(1): p. 157-157.  England | Qualitative | 30 health professionals (midwives, sonographers, obstetricians, anaesthetists) from 2 hospital sites in North-West England. | To understand the health professionals’ experiences and views towards TLC. In addition, the interviews aimed to examine health professionals’ views of the impact of TLC (a 10-week antenatal lifestyle course for women with BMI over 30) on their current clinical practice. | Semi-structured interviews which were audio recorded, transcribed and analysed thematic content analysis. | When discussing PA in relation to weight:   **Weight was felt to be a sensitive topic which made HCPs reluctant to introduce due to risk of causing offence.**  **HCPs felt responsible and that they had to address a large number of issues within a short time.**   **HCPs felt they had to choose their words carefully, and avoiding certain terms that may cause offence in relation to weight.**   **HCPs felt they needed more training and knowledge regarding what advice to give to obese pregnant women.** |  |
| Macleod, M., et al., Provision of weight management advice for obese women during pregnancy: a survey of current practice and midwives' views on future approaches. Maternal and child nutrition, 2013. 9(4): p. 467-472.  Scotland | Cross sectional mixed methods survey- quantitative and qualitative | 78 midwives employed by NHS Tayside | To identify current practice and views on weight management of obese women during pregnancy and the puerperium. | 241 midwives were invited via email to take part in a web-based questionnaire. The questionnaire had questions related to weight management procedures in the antenatal period, perceived barriers to providing weight management guidance, and views on future approaches to address maternal obesity. | **Only 15% of respondents reported offering personalised advice regarding weight management based on physical activity levels.   Less than half (46%) of respondents thought midwives should provide weight management advice to obese pregnant women (including advice on physical activity)   Many midwives reported a shortage of time as a barrier to providing weight management advice.   Midwives are concerned that pregnant women would not be receptive to weight management advice, and were scared of causing fear or embarrassment.** |  |
| Lie, M.L.S., et al., Preventing Type 2 diabetes after gestational diabetes: women's experiences and implications for diabetes prevention interventions. Diabetic medicine, 2013. 30(8): p. 986-993.  England | Qualitative | Phase 1: 31 women with GDM, phase 2: 14 women with GD | To explore factors influencing post-natal health behaviours following the experience of gestational diabetes, and to elicit women's view about the feasibility of lifestyle intervention to prevent diabetes during the first 2 years after childbirth. | Semi-structured interviews with women who had gestational diabetes. Statements were used in Phases 1 and 2 to summarise women's views of lifestyle changes to prevent diabetes and to develop views about diabetes interventions, respectively. | Women were generally appreciative of the **clinical support the received when they had GDM, which helped them to understand the role of physical activity behaviours in controlling their blood glucose during pregnancy**.   Diagnosis of GDM **led to anxiety for the unborn child, and translated into careful concordance with advice about physical activity**. The priority is the baby - and women were strongly motivated to protect their unborn child.   Gaps in women's knowledge about what they should do to reduce their future risk of developing T2D (such as appropriate level of PA) were apparent. **Coupled with attitudes to risk, this led to a disjuncture between women's understanding of future risk and extent to which they engaged in PA**. | **Main barriers to PA were recovery from GDM, tiredness, maternal attachment and demands of childcare.** |
| Arden, M.A., A.M.S. Duxbury, and H. Soltani, Responses to gestational weight management guidance: A thematic analysis of comments made by women in online parenting forums. BMC pregnancy and childbirth, 2014. 14(1): p. 216-216.  UK (national) | Qualitative | Data were collected from three online parenting forums cotaining content on NICE guidance (total of 202 forum members contributing 400 posts). Study included responses / posts from women, healthcare professionals, a journalist and a moderator. All members were women, except for the male television journalist. Members ranged from those trying to conceive, those experiencing pregnancy, and mothers reflecting on past pregnancy experiences. Most women were based within the UK. | To explore women’s perspectives about the weight gain guidance using spontaneous and naturally occurring comments made in posts on public parenting forums. | A thematic analysis of 400 posts made on UK-based parenting internet forums in the week following publication of NICE guidance (which includes advice on healthy eating and physical activity including at least 30 minutes per day of moderate intensity PA). Authors examined naturally occurring comments from 202 women. | There was **confusion** about what the guidance meant in terms of dietary and exercise behaviour, and a **lack of consistency** in how advice and messages are delivered by healthcare professionals. **Women wanted personalised**, **practical advice** delivered **sensitively**. | Some women spoke about other **barriers** in their lives which impacted on their ability to eat well and exercise in pregnancy, for example, tiredness, morning sickness, caring responsibilities and work pressures. |
| Padmanabhan, U., C.D. Summerbell, and N. Heslehurst, A qualitative study exploring pregnant women's weight-related attitudes and beliefs in UK: The BLOOM study. BMC Pregnancy and Childbirth, 2015. 15(1): p. 99-99.  England | Qualitative | 19 pregnant women with different pre-pregnancy BMIs in their third trimester | To explore weight-related attitudes and beliefs during pregnancy (including the weight-related behaviours of diet and physical activity during pregnancy). | Semi-structured interviews which were audio recorded, transcribed and analysed thematic content analysis. | **Women felt their midwives provided detailed information on what they should not do during pregnancy, but were rarely given information about what they should do in relation to physical activity for weight management.**   **Information received from midwives was perceived as "vague", "lacking depth" "insufficient", and focused on restrictions during pregnancy rather than proactive information.**   **Women felt that midwives assumed they already had knowledge about PA.**  **Women preferred being given verbal advice from their midwife rather than assimilating all the written information provided.**   **Most women pondered the benefits of indoor activity and questioned the absence of recommended indoor activities.** | **Women felt that physical activity benefitted them psychologically and physiologically (e.g., helped them relax, enabled easy labour).** **However, they also felt that certain forms of PA could put their pregnancy under severe risk, resulting in prioritisation of good nutrition over physical activity during pregnancy.**   **Diet was considered easier to control than physical activity by pregnant women because of the numerous barriers to physical activity.**  **Women were concerned about the safety of PA, especially as exercises recommended such as walking and swimming needed conducive weather. Most women pondered the benefits of indoor activity and questioned the absence of recommended indoor activities.   Women had concerns around classes and would prefer to attend classes run by midwives or trained professionals.** |
| Heslehurst, N., et al., An evaluation of the implementation of maternal obesity pathways of care: A mixed methods study with data integration. PloS one, 2015. 10(5): p. e0127122-e0127122.  England | Mixed methods, quantitative and qualitative | Study 1: 17 pregnant women (majority in 3rd trimester)  Study 2: Healthcare professionals responsible for delivering the pathways within the NHS Trust (n = 243; 86% midwives and 14% medical clinicians), and there was 68% response | To evaluate the implementation of maternal obesity care pathways from multiple stakeholder perspectives. | This was a simultaneous mixed methods model with data integration. There were 3 component studies (2 relevant for this review). Study 1 involved semi structured interviews exploring obese pregnant women's experiences of being on pathways. Study 2 was a quantitative and qualitative postal survey, exploring HCPs experiences of delivering the pathway. | **Obese pregnant women felt that midwives rarely discussed physical activity.** **Women also stated they wanted more information on pregnancy specific benefits of physical activity.**   When discussing the leaflet specifically, wo**men indicated they liked clear a specific advice relating to exercise (e.g., 20 minutes a day).**   **Midwives indicated that they want training on exercise in pregnancy which would support women's behaviours and access to support services.** |  |
| Dinsdale, S., et al., "as soon as you've had the baby that's it..." a qualitative study of 24 postnatal women on their experience of maternal obesity care pathways. BMC public health, 2016. 16(1): p. 625-625.  England | Qualitative | 24 recent mothers (aged 20-42) living in North East England who had commenced on one of the maternal obesity care pathways through NHS Foundation Trust. 11 had a BMI ≥30/35, and 13 with a BMI ≥40. | To explore accounts of postnatal women who had been through one of these three maternal obesity care pathways in National Health Service (NHS) Foundation Trust in NE England. | Semi-structured one to one interviews (20 in women's homes and 4 via telephone). Interview schedules were designed to explore views and experiences in relation to the pathways. Data were analysed using thematic content analysis. | **Physical activity information was minimal**, and **only provided when women sought this themselves**.   **Women felt midwives may not have time to discuss diet and activity, assuming this was already well understood, and instead focused on other issues e.g., breastfeeding**.   **Women want information about safe activity in pregnancy**   Women felt their understanding of healthy lifestyle during pregnancy should be assessed, **and then information tailored to this as a consequence.**   Women felt information could come from **midwives or via a service specifically designed for pregnant mums, allowing them to address PA in peer settings**.   Women suggested they would find **details of community exercise classes useful.** | **Fears about the safety of the baby** were viewed as a major marrier to participating in physical activity in pregnancy in obese pregnant women. Some described giving up exercise upon becoming pregnant and 'wrapping themselves in cotton wool'.   In obese pregnant women, **extreme tiredness or discomfort** were reported as things preventing engagement in more PA.   **Costs of participating in classes** was also viewed as a barrier.  Exercise classes for pregnant women could provide an opportunity to **socialise and do exercise in a comfortable environment.** |
| Lavender, T. and D.M. Smith, Seeing it through their eyes: a qualitative study of the pregnancy experiences of women with a body mass index of 30 or more. Health expectations : an international journal of public participation in health care and health policy, 2016. 19(2): p. 222-233.  England | Qualitative | 34 pregnant women from two areas in North West England with high concentration of 'more deprived' neighbourhoods. | To gain insight into the experience of pregnant women with BMI ≥30kg/m2 when accessing maternity services and attending a community lifestyle programme. | A general interpretive approach was used to develop an overall picture of women's experiences. 10 focus groups as well as 9 individual interviews (to accommodate more participants) were conducted. | The majority of women in this study reported that **the only advice they were offered was referral to a lifestyle programme**. Whilst receptive to this, they wanted advice from healthcare professionals supporting their care.   Women valued the **'informal style' of those** delivering the programme.   Several women talked of **feeling lonely, and mentioned the programme as a way for them to 'get out once a week'.**   Women reported incorporating PA into daily lives as a result of the information and practical exercise they participated in as part of the programme.   **Learning new forms of exercise and setting personal goals** were facilitators to engaging in PA.   Women felt positively **towards exercises to do with their children**. |  |
| Eades, C.E., E.F. France, and J.M.M. Evans, Postnatal experiences, knowledge and perceptions of women with gestational diabetes. Diabetic medicine, 2018. 35(4): p. 519-529.  Scotland | Qualitative | N=15 women with gestational diabetes mellitus (GDM) recruited from a clinic in one Scottish health board. | To explore experiences, knowledge and perceptions of women with GDM to inform then design of interventions to prevent or delay T2D. | Semi-structured interviews with women recruited from one Scottish health board. A framework approach was used to manage and analyse data according to themes informed by psychological theory. | **Nearly all women in this study recalled being told they were at increased risk of future diabetes and should reduce risk through increasing physical activity levels.**   **Some women recalled being advised to increase activity levels when they were diagnosed, but felt this advice was more brief and peripheral to the education they received on diet**. **Others did not receive any advice**. **There was confusion over what was appropriate in terms of exercise during pregnancy.**   **Women were not satisfied with vague and untailored information, and this led to feelings of frustration**. | **Barriers to PA included: having a bump, back and pelvic pain, demands of having other children, tiredness and poor weather.**   Changes to exercise during pregnancy were often motivation by concern for baby's health. |
| De Vivo, M. and H. Mills, "they turn to you first for everything": Insights into midwives' perspectives of providing physical activity advice and guidance to pregnant women. BMC Pregnancy and Childbirth, 2019. 19(1): p. 462-462.  England | Qualitative | 10 community midwives from 10 randomly selected antenatal clinics. | To gain insights into midwives' perspectives of providing physical activity advice and guidance to pregnant women. | Face-to-face semi structured interviews with community midwives. A combination of inductive and deductive approaches were used to produce a thematic description of the data. | **Midwives perceive themselves to be well placed to provide PA advice and guidance.**   Midwives feel that **pregnant women did not receive adequate information about PA during pregnancy and that the topic was not emphasised enough**.   **Midwives shared frustrations of the buck being passed between exercise and health professionals**. This reluctance of professionals to accept responsibility appears to predominantly affect pregnant women who are **regular exercisers, leaving them without clear guidance and support to enable them to continue PA**.   **PA advice is a 'tick box' exercise, and not explored or revisited unless it is brought up by pregnant women themselves. Only women who are regular exercisers will enquire about PA during pregnancy.**   **Emphasis on PA is consistent with midwives pushing different agendas (dependant on their area of interest or speciality**).   **Lack of training, knowledge and confidence cited as key reasons for not being able to provide effective PA guidance and advice.** Midwives were **unaware of suitable professional resources** (leaflets etc) available to draw upon. Midwives **feel ill-equipped to deliver tailored conten**t, but were happy to signpost to other resources if they had them (e.g., NHS Choices website). **Midwives also had a lack of awareness of local opportunities.** | **Midwives experience increasing demands and expectations   Lack of time in appointments etc leads to a need to prioritise which information is given.** |
| Findley, A., et al., Exploring womens' experiences and decision making about physical activity during pregnancy and following birth: A qualitative study. BMC Pregnancy and Childbirth, 2020. 20(1): p. 54-54.  England | Qualitative | 16 women who were either pregnant or had recently given birth. Participants were of a 'healthy weight' (self-reported pre-pregnancy BMI of 18.5-25). Authors noted reporting on this group as they are often overlooked in research studies which tend to focus on overweight and obesity in pregnancy. 11 were currently pregnant, and 5 had given birth in the last 3 months. | To explore (1) women's experiences of PA during pregnancy and following birth, and (2) decision-making processes related to PA during this time. | Semi-structured interviews (via telephone). An inductive and deductive approach was taken when analysing interviews. The six stages of thematic analysis were used to analyse the data. | Women felt there was a **lack of trusted information available**, with many people (including family and partners) advising women that PA should be stopped during pregnancy, **leading to not knowing whose advice to follow.**  Women reported belief that **being healthy was important during pregnancy and thus had desire to maintain fitness**. Women reported **feeling more energised, and described how PA helped lift their mood and made them feel relaxed**.  Some participants d**escribed how being active made them feel mentally and physically prepared for labour**.  Towards end of pregnancy, **certain activities are seen as 'too intense' e.g., cycling and running**.  Women's pre-existing expectations of the **level of PA they would be able to achieve during pregnancy was influenced by knowledge of how active other pregnant women had bee**n, **as well as advice provided by healthcare professionals**.  Women felt they were entering unknown territory, experiencing conflict between wanting to be active but wanting reassurance the baby was healthy. Being unable to monitor baby's health made **PA feel like a risky behaviour**. **Women felt they would rather have time off exercise and know that the baby is fine**.  **Over half of participants gained variable information from HCPs during pregnancy and following birth**.  Most women **wanted advice that was evidence-based or from professionals who were trained** to know **what constitutes safe PA while pregnant**.  Around **half reported advice they received to be unclear or conflicting in nature, leaving them feeling alone and left to use common sense**.  Guideline knowledge was low.  Some women felt advice received to be overly cautious as a result of the HCP feeling personally liable if something bad happened.  Participants felt advice **was not tailored to their personal circumstances and was more risk averse than necessary, leading to it being ignored.** | Women felt that whilst others had tried to offer advice, they felt it was important to listen to their own body.   Women felt like some advice about resting etc was unsolicited, and for example "I stayed quite active because I felt fine, and I don't want to be resting all the time"  **Stigma / misconception that PA is dangerous during pregnancy (e.g., people at work and friends / family).**   **Women who were more active before pregnancy were more likely to be active during pregnancy**.  **Morning sickness, fatigue, aches and pains are physical limitations to being active during pregnancy. Some women perceived these as signs that they were pushing too hard, potentially causing harm to themselves or the bab**y.   **Women felt a strong sense of social pressure to conform to other people's views of PA during pregnancy**.   **Changing body shape changed behaviour - women avoided certain PA to avoid bumping their bump.** |
| Lawrence, W., et al., How can we best use opportunities provided by routine maternity care to engage women in improving their diets and health? Maternal and child nutrition, 2020. 16(1): p. e12900-n/a.  England | Secondary data analysis of qualitative and quantitative data | Face-to-face Interviews: 20 women from HCS and 10 women from control   Focus groups: 4 HCS HCPs  Case report: 20 women interviewed from HCS  Telephone interviews: 17/20 women from HCS | To explore the experiences of pregnant women and research midwives/nurses of a brief intervention called Healthy Conversation Skills (HCS) being delivered as part of a randomised control trial, assessing the acceptability and feasibility of including this intervention in routine maternity care. | Analysed data from the SPRING trial (Southampton Pregnancy Intervention for the Next Generation) - a randomised controlled trial of vitamin D supplementation alongside brief intervention that aims to improve pregnant women's diet and PA levels. The intervention is delivered via HCS-trained midwives. In this study, qualitative data sets and quantitative data sets were analysed. Qualitative data were analysed thematically. | Some women reported **feeling judged or under pressure to make a change**.   **Midwives were concerned about how participants may react to attempts to engage them in discussions around PA as this is typically seen as a difficult issue to raise**,but found that on reflection the concerns were unfounded.   Women in the intervention arms described being prompted to identify barriers to change and strategies for overcoming these, like taking alternative forms of exercise.   Women **stated a desire for NHS midwives to ask questions to explore their perspectives rather than be left to take responsibility for raising issues**. For example, **"it's always left to the women to ask the midwife like 'can i do more exercise?' rather than the midwife instigating it**..." |  |
| De Vivo, M. and H. Mills, Laying the foundation for pregnancy physical activity profiling: A framework for providing tailored physical activity advice and guidance to pregnant women. International journal of environmental research and public health, 2021. 18(11): p. 5996.  England | Cross-sectional, quantitative | N=89 pregnant women attending NHS antenatal clinics | To examine the predictive utility of the theory of planned behaviour in explaining pregnant women's physical activity intentions and behaviour, and to scrutinise the role of past behaviour within this context. | Participants completed the pregnancy physical activity questionnaire and newly developed theory of planned behaviour questionnaire on two separate occasions during pregnancy. Analyses were carried out in relation to three scenarios. | **Intention emerged as the strongest determinant of pregnant women's PA behaviour.   Controlling for past behaviour attenuated the influence of intention and perceived behavioural control on behaviour, with neither of the original variables providing a unique influence.**   The addition of past behaviour added significantly to the prediction of intention with the model as a whole, explaining 85% of the variance in pregnant women's PA intention, and with past behaviour uniquely contributing 44.8% of the variance.   Pregnancy PA profiling based on intention and behaviour status is introduced here as a novel and practical framework, providing HCPs with the opportunity to structure and provide tailored advice and guidance to pregnant women. **Specifically, four profile types are being proposed: (1) women who have been regularly active in the past and intend to continue to be throughout pregnancy, (2) women who have been active in the past and do not intend to continue to be, (3) women who have been inactive but intend to be during pregnancy, and (4) pregnant women who have been inactive and don't intend to become active during pregnancy. Strategies such as brief advice may be more suited to the first group, person centred approach may be more suited for group 2, motivational interviewing may be required for group 4 etc.** |  |
| Ells, L., et al., Consistent delivery of healthy weight messages to pregnant and postpartum women: A local resource implementation evaluation commissioned by Public Health England 2018, Teesside University.  England | Qualitative | HCPs (including midwives and health visitors) from Manchester and North Yorkshire took part in workshops (n not known). Online surveys were completed by 5 participants and 4 took part in interviews (health care workers). | To help develop and evaluate approaches that support the systematic and consistent delivery of healthier weight messages in two local pilot areas. | Two local areas were selected. Local implementation and development workshops were held within each of the two pilot areas with local public health midwives, health visitors, and early year staff. Each pilot area was then tasked with developing and delivering their local implementation plan. Manchester produced a maternal postnatal health record, and North Yorkshire provided a set of healthy messaging resources to HCPs. An online survey, co-developed with workshop attendants, was used to capture learning around the use, implementation, and potential impact of locally developed resources. Further insights were gained from in-depth interviews with key stakeholders from each site. | **HCPs lacked training, time and supporting resources, and felt fear of upsetting patients prevented them from delivering messages about healthy weight (physical activity and healthy eating) to pregnant women.   Participants felt resources should be more visual to help overcome literacy issues, and that illustrations should provide an inclusive representation of the populations served locally.** |  |
| **Key for coding:** | | | | | **Confusion / lack of clarity  Lack of consistency  Importance of personalisation / targeting / tailoring Tone / choice of words  Barriers to PA  Not all pregnant women receive advice on PA during pregnancy Amount and type of information being delivered Type of information desired  Challenges to PA communication by HCPs in practice  Important PA messengers Perceived benefits of PA in pregnancy  Stigma that PA is dangerous What pregnant woman want from PA Facilitators to PA Delivery preferences  Guideline knowledge *Other information sources (non-HCP)* Perceptions of PA** | |
